# Water-Rock Interaction Processes: A Local Scale Study on Arsenic Sources and Release Mechanisms from a Volcanic Rock Matrix

**DOI:** 10.3390/toxics10060288

**Published:** 2022-05-27

**Authors:** Daniele Parrone, Stefano Ghergo, Elisabetta Preziosi, Barbara Casentini

**Affiliations:** Water Research Institute—National Research Council, IRSA-CNR, Via Salaria km 29.300, PB 10, 00015 Rome, Italy; stefano.ghergo@irsa.cnr.it (S.G.); elisabetta.preziosi@irsa.cnr.it (E.P.); barbara.casentini@irsa.cnr.it (B.C.)

**Keywords:** potentially toxic elements, aquifer, volcanic tuff, sequential extraction, Fe oxyhydroxides, sorption

## Abstract

Arsenic is a potentially toxic element (PTE) that is widely present in groundwater, with concentrations often exceeding the WHO drinking water guideline value (10.0 μg/L), entailing a prominent risk to human health due to long-term exposure. We investigated its origin in groundwater in a study area located north of Rome (Italy) in a volcanic-sedimentary aquifer. Some possible mineralogical sources and main mechanisms governing As mobilization from a representative volcanic tuff have been investigated via laboratory experiments, such as selective sequential extraction and dissolution tests mimicking different release conditions. Arsenic in groundwater ranges from 0.2 to 50.6 μg/L. It does not exhibit a defined spatial distribution, and it shows positive correlations with other PTEs typical of a volcanic environment, such as F, U, and V. Various potential As-bearing phases, such as zeolites, iron oxyhydroxides, calcite, and pyrite are present in the tuff samples. Arsenic in the rocks shows concentrations in the range of 17–41 mg/kg and is mostly associated with a minor fraction of the rock constituted by FeOOH, in particular, low crystalline, containing up to 70% of total As. Secondary fractions include specifically adsorbed As, As-coprecipitated or bound to calcite and linked to sulfides. Results show that As in groundwater mainly originates from water-rock interaction processes. The release of As into groundwater most likely occurs through desorption phenomena in the presence of specific exchangers and, although locally, via the reductive dissolution of Fe oxy-hydroxides.

## 1. Introduction

Arsenic is a natural element easily found throughout the environment. Its presence in drinking water represents a hazard to human health [1]. Long-term exposure to As-rich waters can cause serious diseases, from skin lesions, cardiovascular diseases, and type II diabetes to bladder, lung, and skin cancers [1,2,3]. In groundwater, this element frequently shows concentrations exceeding 10.0 μg/L, which is the guideline value suggested by WHO [4] for drinking water and was implemented in Europe as standard by 98/83/EC [5], entailing a prominent risk to human health.

The presence of this potentially toxic element (PTE) in groundwater depends on the hydrogeological setting as well as on various natural processes, including climate, biological activity, and volcanic emissions. Since the 1990s, wide occurrences of As in well water in Bangladesh have attracted the attention of the scientific community, which seeks to deepen its knowledge of the origin and fate of arsenic in groundwater [6,7,8,9]. Water-rock interactions under favorable biogeochemical conditions are considered to be the most important mechanism for the release of As in aquifers by far [10]. Arsenic concentrations in natural waters may range from 0.5 to > 5000 μg/L [9]. Arsenic-enriched groundwater has been reported in many parts of the world, mainly in Asia (Bangladesh, China, India, Vietnam, Nepal), the Americas (USA, Mexico, Argentina, Chile, etc.), and Europe (Italy, Greece, the Pannonian Basin, etc.) [11]. Arsenic release in groundwater has been mainly attributed to three possible processes: the reductive dissolution of Fe/Mn hydroxides, ion exchange with different competitive exchangers (e.g., phosphate, bicarbonate, and silicate), and the oxidation of arsenic-bearing minerals [9,12,13]. Precipitation/dissolution, sorption/desorption, biotic and abiotic oxidation/reduction [14,15], and complex formation are the main reactions controlling the distribution of arsenic between the aqueous and solid phases.

The scientific literature is abundant regarding the presence of As and the main processes leading to its release in groundwater in alluvial environments [16,17,18,19,20], though less so in volcanic contexts. Alarcón-Herrera et al. [21] found a co-occurrence of As and F in oxidized and alkaline environments due to interactions with volcanic glass and, to a lesser extent, with hydrothermal minerals. Further, they identified Fe, Mn, and Al (hydr-)oxides as secondary sources due to their great adsorbing properties. In Argentina, Francisca and Carro Perez [22] reported the presence of As, F, and V in groundwater due to volcanic glass leaching. In a volcanic aquifer in Mexico, under oxidative conditions and high temperature, Morales et al. [23] observed an As mobilization from metallic sulfides, which was favored by thermal water rising through faults and fractures. Ren et al. [24] identify Tertiary volcanic tuffs as the main source of the high As content in groundwater, hypothesizing an origin linked to the leaching of Arsenogoyazite-like arsenic minerals. In a geothermally active area of northern Greece, Winkel et al. [25] analyzed different travertines containing high levels of arsenic (up to 913 mg/kg) and found that it mainly coprecipitated with calcite. In this regard, Di Benedetto et al. [26] claimed that As incorporation in calcite may be an effective limit of As mobility under conditions where immobilization through sorption by Fe and/or Mn oxy-hydroxides is not acting.

In the volcanic aquifers of northern Latium (central Italy), the presence of As and other PTEs typical of the volcanic environment is well known [27,28]. However, the main origin of the As contamination, as well as whether a common process associates arsenic and the other PTEs, is still unclear. Vivona et al. [29] claimed volcanic rock leaching in cold groundwater, while Angelone et al. [30] pointed to a major influence of localized thermal fluids rising along fractures. In the same region, Armiento et al. [31] and Cinti et al. [32] ascribed the diffused As concentration in groundwater to water-rock interaction processes, locally enhanced by thermalism and volcanic gas emissions. Casentini et al. [33] performed batch leaching tests to investigate the release of As induced by interactions of volcanic rocks (lava and tuffs) with inorganic anions and found a positive influence of F and HCO_3_, likely due to anion exchange processes.

The main laboratory experiments aiming to understand the mechanisms of arsenic release include column tests [34,35,36] and batch tests [37,38,39]. Several laboratory studies showed that As is released from sediments under anaerobic conditions [40,41,42], particularly following the reductive dissolution of Fe and Mn oxyhydroxides [43]. Batch tests are often used to study the effects of oxidation-reduction potential (ORP) variations [19], which also affect pH [44] and could naturally occur as a consequence of the mixing of waters with different chemical compositions. Frohne et al. [38] found that dissolved As concentration decreases significantly with increasing ORP and concluded that low ORP levels promote As mobility. Batch tests have also been used to investigate As sorption and dissolution kinetics linked to various mineral phases [45,46].

Arsenic is relatively scarce in the Earth’s continental crust (1.5–2 mg/kg) and can be associated with different As-bearing phases [47]. In this regard, Selective Sequential Extraction (SSE) is a valuable procedure to quantify the distribution of As between different solid fractions that constitute the rocks [48,49,50,51], potentially providing important information about the adsorption-bearing phases and related processes occurring at the soil/water interface [52]. The literature does not lack works on sequential extraction methods aimed at arsenic fractionation [53,54,55,56,57,58,59], and additional procedures have been proposed to improve the extraction of specific As-bearing phases [60,61]. SSE has been used to characterize As distribution and mobility both in contaminated areas [62,63,64,65] and in pristine aquifers.

This work aims to study the main mechanisms that govern As behavior in the water-rock interaction processes in a volcanic context where aquifer rocks are dominated by tuffs. The research was organized in the following three phases:

- Sampling and analysis of water samples from 69 private wells and springs tapping a volcanic aquifer located just north of Rome for the geochemical characterization of groundwater.
- Selective Sequential Extraction operated on selected tuff samples extracted from three quarries within the study area for defining the As distribution among the different solid phases that potentially constitute the aquifer matrix.
- Two batch tests realized on one of the samples analyzed by SSE in order to simulate water-rock interaction processes in different conditions.

Lastly, field and laboratory data have been analyzed in order to evaluate the significant connections between aquifer mineralogy and groundwater geochemistry, with particular reference to As, and also to deepen its relationships with other PTEs in groundwater.

## 2. Materials and Methods

### 2.1. Study Area

**Figure 1:**
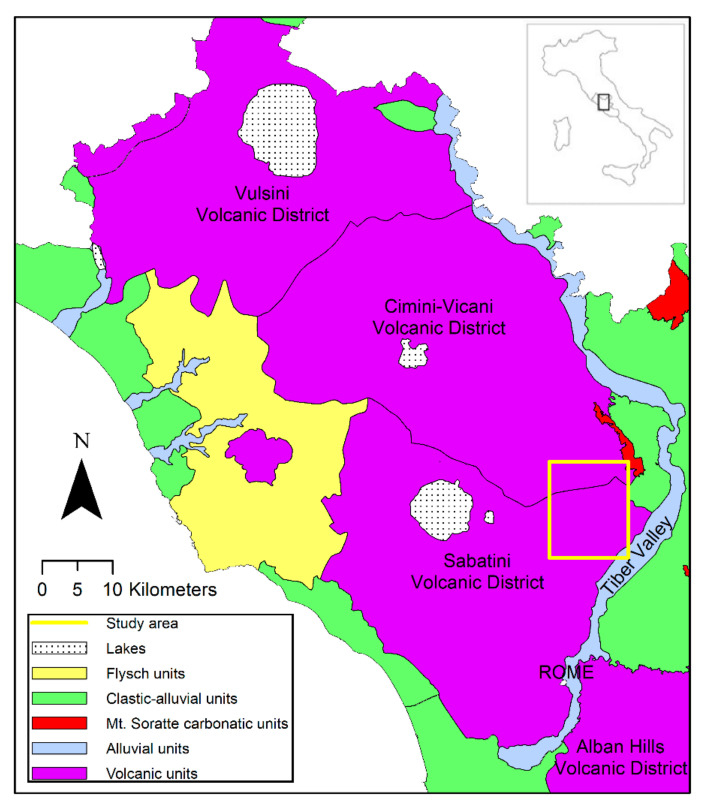
Hydrogeological scheme of Latium volcanic domain, from Parrone et al. [66]. The yellow box indicates the investigated area.

The study area (Figure 1) lies on the eastern flank of the Sabatini volcanic apparatus.

Its activity developed from 0.6 to 0.04 M.a. b.p. through five main phases, during which ultrapotassic volcanic rocks ranging from trachybasalts to trachytes to phonolites were erupted. During the Sabatini volcanic district formation, the Bracciano lake volcano-tectonic depression and several calderas developed [67,68,69]. The volcanic activity developed through several vents distributed over a large area, the main one being the Sacrofano system in the east and the Bracciano system in the west.

Pleistocene volcanic products, which are usually characterized by an overall medium permeability [70], overlap, in angular unconformity, Pliocene and Pleistocene sedimentary deposits of marine and continental origin along with clays and silts that form the low permeability bottom of the groundwater body.

**Figure 2:**
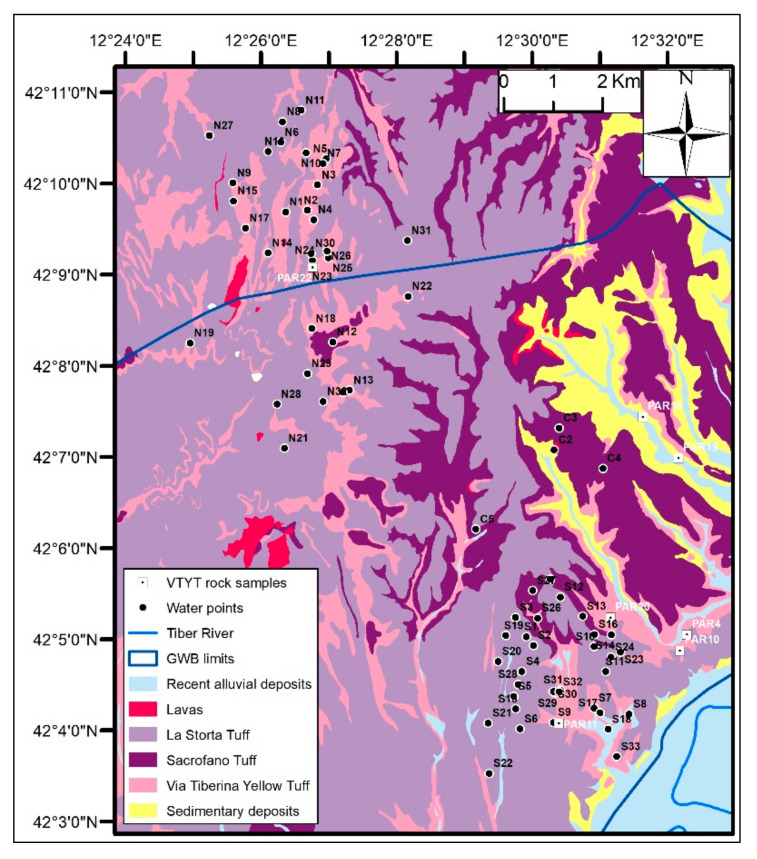
Simplified geological scheme with the location of the rock sampling points of the “Via Tiberina Yellow Tuff” (VTYT) and the groundwater sampling points.

The volcanic deposits outcropping in the study area include mainly pyroclastic deposits: “Via Tiberina Yellow Tuff” (VTYT), “Sacrofano Tuff”, and “La Storta Tuff” (Figure 2). Among these, the VTYT is the most important formation in the investigated aquifer. Cineritic levels and paleosoils determine local decreases of the vertical permeability resulting in a multilayer aquifer with semiconfined horizons [71].

The VTYT is a pyroclastic flow (ignimbrite) produced by the activity of the Sacrofano center, located about 15 km west of the Tiber Valley, which has been set in place directly on the Plio-Pleistocene sedimentary basement. The VTYT has several overlapping units with different lithological characters. Nappi et al. [72] identified a stratigraphically upper lithology with components of smaller dimensions and, between the abundant zeolites, chabazite prevailing over phillipsite; the other, which can be found in the lower levels of the formation, has coarse grain size, has more abundant phillipsite, and shows alterations (clays) due to the action of groundwater. De Rita et al. [73] distinguish three units, spaced by pyroclastite layers of different nature, while Campobasso et al. [74] recognize at least seven depositional units.

The formation has been extensively described from the petrographic and chemical points of view by Lombardi and Marra [75]. The VTYT appears as a massive aggregate, mainly lithoid, of pumices of centimetric sizes linked by a very fine matrix, with colors ranging from yellow to light gray. The average porosity stands at 48% [75]. As reported by Jackson et al. [76], glassy matrix represents 42–54% of the rock, lithic fragments and crystals 6–14%, and secondary minerals such as zeolites and calcite 37–48%. Zeolitization processes of the vitreous mass are evident, with the formation of chabazite and phillipsite aggregates that are primarily responsible for the high degree of lithification of the rock. Another widespread secondary mineral is calcite, resulting from precipitation of the water circulating in diagenetic phases and present not only in the common carbonate fragments but also in the cementing matrix [75].

### 2.2. Groundwater Sampling and Analysis

The first phase of the work consisted of sampling and analysis of groundwater samples from the investigated area. This phase is aimed at the geochemical characterization of circulating groundwater, with a particular focus on the As content and distribution in the two study sectors.

A total of 69 groundwater samples (Figure 2) were collected from wells (64) and springs (5), of which 32 were in the northern sector and 37 were in the southern one. These are mainly private wells for domestic/irrigation use.

At each site, pH, EC, Eh, DO, and water T were measured with probes (Hach-Lange). The sampling was carried out after a purging of the wells until the physical-chemical parameters stabilized (usually 20–30 min). All samples were filtered in the field with 0.45 µm membrane filters under N_2_ and stored in HNO_3_ 1% rinsed polyethylene bottles. One fraction was immediately acidified by HNO_3_ (Suprapure, Merck) for major cations and trace metal determination. Bicarbonates were determined in laboratory by HCl (Suprapure, Merck) titration on 50 mL of sample within 24 h from sampling. Field blanks with ultrapure water were periodically collected, checking for possible environmental contaminations. The samples were analyzed for anions by ion chromatography (IC, Dionex DX-120), for major cations by ICP-OES (Perkin Elmer P400) and trace elements by inductively coupled plasma mass spectrometry (ICP-MS, Agilent 7500c); certified materials (NIST 1640a, trace elements in natural waters) were used to check accuracy of the laboratory measurements. The electro-neutrality (EN%) evaluated as the percent difference for major cations and anions, including F and NO_3_, ranges between −3.96% and +5.10%. For statistical analysis, the concentrations below detection limit (LOD) were assumed to be equal to half of LOD itself.

### 2.3. Statistical Analysis of Groundwater Chemical Data

A univariate statistical analysis of groundwater chemical data was carried out. Groundwater analytical data were processed by means of descriptive basic statistics, and the concentrations of some parameters were represented via scatterplots and distribution maps.

Normality or lognormality of As distribution was verified using the Shapiro-Wilk goodness of fit test [77]. In addition, Rosner’s parametric test [78] and Huber’s non-parametric test [79] for detection of data outliers were applied.

Finally, a bivariate correlation analysis in the two study sectors was carried out using both Pearson’s linear correlation index, which is useful in case of normal data distributions, and Spearman’s rank correlation index, which is more robust and suitable for skewed distributions or in presence of data outliers.

Data processing was performed through different software: ProUCL 5.1 [80] for the normality/lognormality and outlier detection statistical tests, Past 4.05 [81] for the bivariate correlation analysis, and ArcGIS 10.2.2 for the As concentration mapping.

### 2.4. Outcropping Rock Sampling and Mineralogical Analysis

Seven rock samples from the “Via Tiberina Yellow Tuff” (VTYT) were collected for mineralogical analysis (Figure 2). Four of these samples (PAR04, PAR10, PAR11, and PAR22) were extracted from excavation fronts of VTYF tuff quarries. The superficial portion of the samples was removed in order to eliminate the part of the rock that has undergone exogenous alteration processes. The samples were then transported to the laboratory for the analysis.

Tuff samples were analyzed by X-Ray Powder Diffraction (XRPD) and Scanning Electron Microscopy (SEM), in order to have a first mineralogical characterization of the aquifer matrix. The XRPD patterns were collected with a Siemens D5000 diffractometer operating with Bragg-Brentano θ/2θ geometry, CuKα = 1.518 Å, 40 kV, and 40 mA. Each XRPD pattern was collected from 4 to 80° of 2θ, with a step scan of 0.02°. Identified Bragg reflections were assigned to the corresponding crystalline standards contained in the inorganic crystal structure database (ICSD). Powdered samples were prepared by hand grinding using an agate mortar and pestle, then sieved with a sieve of 0.5 mm mesh. Powders were further ground prior to the analysis to achieve a particle size < 50 µm.

SEM observations were performed with the Philips XL30 Analytical Scanning Electron Micro-scope equipped with secondary (2 nm imaging resolution), backscattered (0.1 AZ elemental resolution) electron detectors, and probe for Energy Dispersive X-ray Analysis (EDAX 134 eV) for the execution of punctual elemental analysis of the mineralogical phases and spectrum representation. SEM investigations were carried out on ordinary 30 µm thin sections after graphite sputter-coating of the samples.

Preliminary observations of the thin sections at the optical polarizing microscope (Nikon Eclipse E400 Pol) were also performed.

### 2.5. Selective Sequential Extraction

For three selected VTYT samples (PAR10, PAR11, and PAR22) we performed a Selective Sequential Extraction (SSE) in order to determine the distribution of As between the different solid bearing phases forming the rocks and to better understand the possible processes governing As mobility. Several protocols are available in literature to perform SSE analysis [82,83]. For the extraction procedure, we chose the methodology proposed by Wenzel [57] and modified it in order to consider a specific step for calcite from Costagliola et al. [61] and one for sulfides from Torres and Auleda [84].

The final procedure is able to distinguish seven fractions that can potentially bind As: (1) easily exchangeable As, (2) specifically adsorbed As, (3) As bound with calcite, (4) low crystalline Fe(III)-oxyhydroxides, (5) crystalline Fe(III)-oxides, (6) sulfides, and (7) residual fraction. The seven different steps that constitute the procedure are presented in Table 1.

For the SSE, we started from 1 g of tuff powder. After each step, the suspension was centrifuged at 5000 rpm for 10 min, supernatant separated, diluted with MilliQ (18.2 MΩ cm at 25 °C), acidified with 1% HNO_3_, and stored at 4 °C until analysis conducted by ICP-MS (Agilent 7500c). Two replicates were performed for each rock sample.

### 2.6. Batch Tests

In order to investigate the water-rock interaction processes and evaluate the release of arsenic and other elements under different conditions, two batch tests were performed on a previously selected Yellow Tuff sample (PAR11). In polyethylene bottles, two different extracting solutions (40 mL) were added to 2 g of pulverized rock (solid/liquid ratio 1/20) using:

- Synthetic water consisting of MilliQ (18.2 MΩ cm at 25 °C) to which KNO_3_ was added until obtaining a low ionic strength solution (0.5 mM) to simulate contact with rainwater.
- As-free groundwater with ionic strength and physical-chemical characteristics similar to those of groundwater circulating in the study area, simulating the water-rock interaction processes.

The chemical composition of the two extracting solutions is reported in Table S1.

The bottles were placed in a horizontal shaker at 400 rpm at room temperature, and aliquots (20 mL) were taken at fixed intervals (3, 6, 12, 24, 48, 120, 720 h). Samples were centrifuged at 5000 rpm for 10 min and supernatant separated, diluted with MilliQ (18.2 MΩ cm at 25 °C), acidified 1% with HNO_3_, and stored at 4 °C until analysis. Before each sampling step, pH value of the samples was measured with probes. After each sampling, 20 mL of fresh solution was added to the bottles, inducing new dissolution at each step.

A second test was carried out with an experimental apparatus consisting of a 500 mL polyethylene bottle filled with the solid matrix (PAR11) and the synthetic rainwater. Oxygen, redox, and pH variations in the system were continuously monitored by DO, Eh, and pH electrodes. The system was also equipped to be N_2_ fluxed, and water samples were collected at fixed intervals. In addition, a solution of sulfide (25 mg/L S^2−^) was injected into the batch solution to simulate sulfide-rich hypoxic conditions. A total of 20 g of the pulverized tuff were placed in 400 mL of synthetic solution and stirred for the whole duration of the test using a magnetic stirrer. The test was divided into different steps:

- STEP 1 (dissolution under aerobic conditions): The sample was in solution under oxygenated conditions. We carried out water sampling after 0, 4, 24, 48, 72, and 96 h.
- STEP 2a (induced anaerobic conditions): subsequently, the test continued by blowing N_2_ within the system in order to eliminate the oxygen present. In this phase, we collected water samples after 24, 48, and 72 h.
- STEP 2b (induced anaerobic condition with sulfide presence): After repeating STEP 1 (4 days in oxygenated conditions with no sampling), the test continued under N_2_ flux and adding sulfide to reach 0.5 mg/L final concentration. We then collected aliquots at 0, 3, 6, 24, 48, and 72 h.

For each sample, 15 mL of water was collected with a syringe, filtered with 0.4 μm (polycarbonate filters), diluted with MilliQ (18.2 MΩ cm at 25 °C), acidified by 1% with HNO_3_, and stored at 4 °C until analysis. Samples of the two batch tests were analyzed for anions (only filtered aliquots) by IC (Dionex DX-120), for Fe by UV/Vis spectrophotometry (Hach Lange DR2800), and for trace elements by ICP-MS (Agilent 7500c).

## 3. Results and Discussion

### 3.1. Groundwater Geochemistry

As observed by Parrone et al. [66], piezometric levels decrease according to the main flow direction from NNW-SSE towards the Tiber River. Groundwater shows the dominance of a bicarbonate-earth-alkaline facies, with samples ranging from Ca-HCO_3_ types to Na+K-HCO_3_ types.

**Figure 3:**
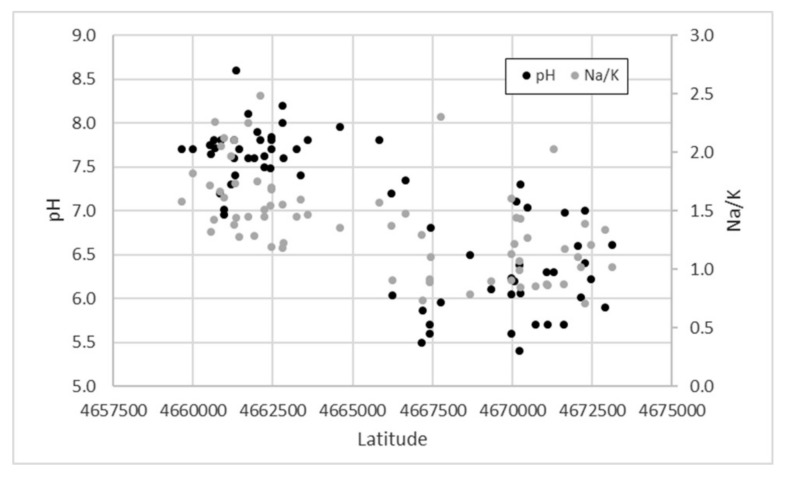
Latitude trend of the pH values (left ordinate scale) and the Na/K ratio (right ordinate scale).

Water samples clearly show different pH values, passing from acidic (median = 6.1) to alkaline (median = 7.7) conditions from the northern to the southern sector (Figure 3). Low pH values in the north are probably related to a widespread circulation of CO_2_ that can be hypothesized to be in the area of the hydrogeological watershed near the ancient Sacrofano caldera, as evidenced by the bubbling observed during the sampling of numerous wells. Some parameters typical of volcanic environments (e.g., PO_4_, V), together with Al, show higher concentrations in the northern area, while an enrichment in HCO_3_ and Na can be observed in the south (Table S2). This suggests a greater influence of the sedimentary deposits on the groundwater chemistry in the southern sector. As shown in Figure 3, in support of this hypothesis, the Na/K ratio increases from the north (median = 1.05) to the south (median = 1.55). The enrichment in K represents a typical feature of the waters circulating in the potassium-alkaline volcanites of Central Italy [29,85].

Arsenic does not present a clear spatial distribution (Figure S1), showing slightly higher values on average in the southern area and some localized peaks. In most of the samples (91.3%), As exceeds the drinking water standard (10.0 µg/L), without particular differences between the northern (maximum value 50.6 µg/L and 90.6% of exceedances) and the southern sector (maximum value 50.2 µg/L and 91.9% of exceedances).

In both sectors, As shows lognormal distributions (Shapiro-Wilk test, α = 0.05) and no data outliers (Rosner’s parametric test, Huber’s non-parametric test).

In Table 2, As’ correlation with the chemical and physical-chemical parameters for the two sectors is shown. In the northern area, As shows direct correlations, both parametric and non-parametric, with F, U, and V. In the southern sector, the strong positive correlation with F remains along with Na, K, Li, and B, while a significant inverse correlation with oxygen can be observed.

### 3.2. Mineralogical Characterization of the Rocks

The VTYT looks generally massive and coherent, with a yellow-gray color. The formation is usually affected by significant subvertical fractures and is often mineralized. Macroscopically, the tuff appears as an aggregate of pumice embedded in an ashy matrix. At the microscopic level, the VTYT samples show different fragments of rocks and minerals, such as sanidine, plagioclase, pyroxene, leucite (often analcimized), biotite, garnet, and apatite. The tuff skeleton also presents fragments of carbonate rocks, presumably ripped from the basement during the eruption.

XRPD highlighted the constant and important presence of zeolites (chabazite/hershcelite), a result consistent with data previously reported in the literature about this type of tuffs [75]. Other minerals identified within the rock with this technique include calcite, quartz, K-feldspar, mica (biotite), plagioclase, kaolinite, and chlorite.

**Figure 4:**
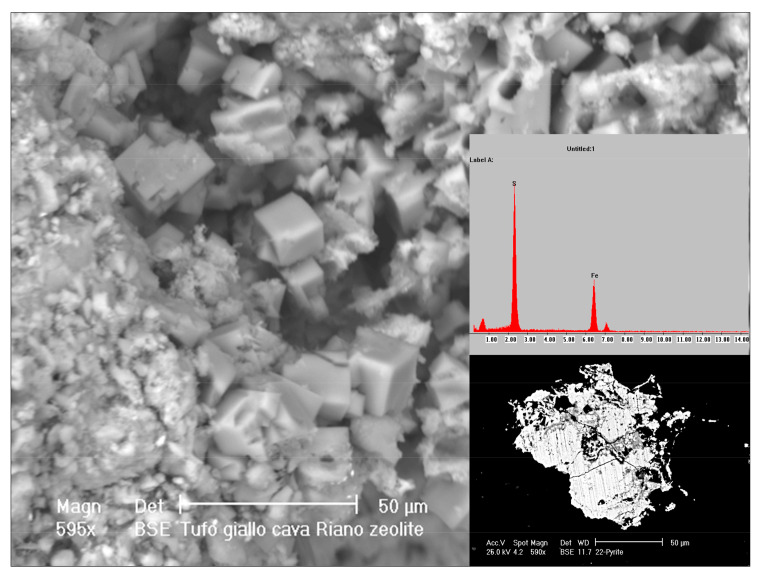
SEM images: zeolites and pyrite (right) within the Via Tiberina Yellow Tuff. For pyrite the elemental analysis is also shown.

SEM-EDX elemental analysis allowed us to identify other minerals within the rock: pyrite (Figure 4), magnetite, secondary calcite, titanite, rutile, zircon, monazite, other Fe, and rare earth oxides.

One of the objectives was the research of possible mineral phases containing particularly relevant As concentration; however, the qualitative analysis of the investigated crystalline forms showed no traces of elevated As, suggesting its possible presence as homogeneously dispersed arsenic at a level below the LOD of this technique.

### 3.3. As Fractioning within the Selected Tuffs

We performed a Selective Sequential Extraction on three selected VTYT samples (PAR10, PAR11, PAR22), with the purpose of quantifying the total As content and its distribution among the different solid phases that potentially constitute the aquifer matrix. The results allowed us to hypothesize the As-bearing phases that are more easily mobile from circulating waters and, accordingly, are the most likely geochemical mechanisms that can cause arsenic release from the investigated volcanic matrix.

**Figure 5:**
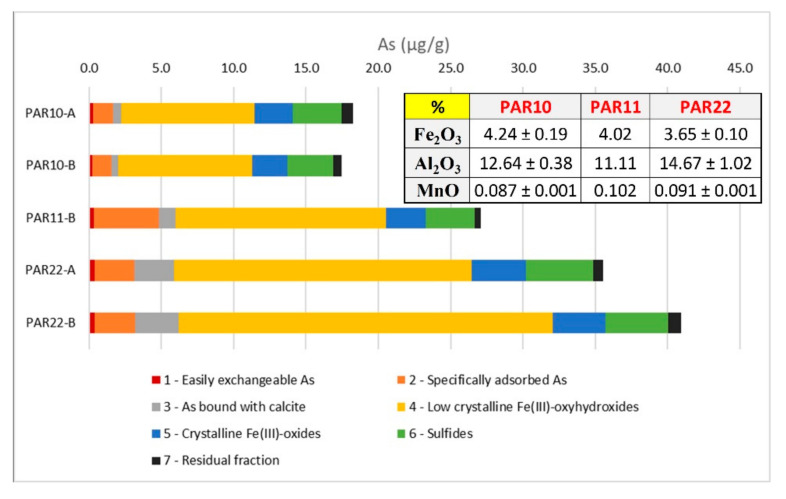
As fractioning resulting from the sequential extraction (two replicates for PAR10 and PAR22). The table (right) shows the weight percentage of interesting oxides in the rocks (mean ± std. dev. for PAR10 and PAR22).

The total arsenic in the sampled rocks is in the range of 17.5–40.9 mg/kg, which is data that is consistent with the findings of Armiento et al. [31] regarding the deposits of the Cimino-Vicano volcanic district. The sequential extraction procedure (Figure 5) shows As distribution among different solid fractions.

The As found in easily exchangeable fraction is negligible (0.22–0.41 mg/kg, corresponding to 0.9–1.4% of the total As). Conversely, the arsenic specifically adsorbed onto mineral surfaces ranges from 1.31 to 4.53 mg/kg (6.9–16.7%). Competitive ions in solution (e.g., phosphate) can affect the mobilization of this fraction, which can thus play an important role in the As contamination of groundwater in the study area.

Arsenic association with calcite can be significant (0.52–2.97 mg/kg, 3.4–7.8%), and the calcimetry measurements showed a total content of CaCO_3_ between 5.5% and 12.0%. As stated by Alexandratos et al. [86], the mechanisms of arsenate sorption on the calcite surface and of its incorporation in the calcite lattice are very similar to the point where surface sorption can be considered to be a precursor of incorporation in the crystal lattice; therefore, it may be difficult to distinguish between adsorbed and incorporated As(V) complexes at the calcite surface. As a result, at least part of the specifically adsorbed As extracted in step 2 is likely derived from As incorporated in the lattice at the calcite surface.

In this step, the acetic buffer is used to selectively dissolve the carbonate, but Fe-oxyhydroxides may re-adsorb the As oxyanions liberated by the calcite dissolution. At pH 5 (acetate buffer), the capacity of Fe-oxyhydroxides to adsorb As-oxyanions is high, thus leading us to underestimate the amount of As bound to calcite, and conversely, to overestimate As bound to Fe-oxyhydroxides in the subsequent step. This effect cannot be quantitatively assessed, and therefore, the amount of As bound to calcite recovered by the specific step must be considered as a minimum estimation [61]. A release of As linked to calcite in groundwater of this area cannot be excluded, particularly where favorable conditions for dissolution exist (e.g., acidic conditions in the northern sector near the hydrogeological watershed).

Most of the As (11.69–29.51 mg/kg, 63.8–72.1%) that was extracted during the procedure is linked to Fe oxy-hydroxides, especially low crystalline (50.2–63.2%), which nevertheless constitutes only about 4% of the solid matrix. Commonly, amorphous phases present a larger specific surface area [87] than crystalline structures and tend to also adsorb As within their loose and hydrated structures, not only on their outer surfaces [88]. Furthermore, the conversion of amorphous ferrihydrite to crystalline Fe-oxide phases, which may gradually occur over time [89], can reduce the density of As sorption sites [90,91,92], leading to the desorption of adsorbed As [93]. These considerations are relevant in the study of As release for the dissolution of amorphous and crystalline phases in presence of reducing conditions of the aquifers [94]. Although oxidizing conditions are largely dominant in the study area, local conditions potentially favorable to the reductive dissolution of iron oxyhydroxides can also occur, with the consequent possible release of associated As.

Arsenic’s association with sulfide fractions (3.17–4.64 mg/kg, 10.7–18.4%) confirms the important presence of sulfide minerals, such as pyrite, which was already observed during the SEM observations. Smedley and Kinniburgh [9] reported this association, emphasizing the chemical closeness of As and S and the occurrence of the highest As concentrations in sulfide minerals, among which pyrite is generally abundant in volcanic and geothermal contexts. It is, however, an As fraction hardly mobilized in the normal conditions of groundwater of the study area, which are always strongly undersaturated with respect to As sulfides.

### 3.4. Batch Experiments

Two batch tests were realized on one of the samples (PAR11) characterized with SSE. The first experiment, simulated rainwater-rock and groundwater-rock interaction processes to evaluate the As release from the tuff in contact with solutions of different ionic strength. The second experiment provided information about the influence that pH and redox potential can exert on the As-release dynamics and allowed us to investigate processes that may naturally occur in the aquifer.

**Figure 6:**
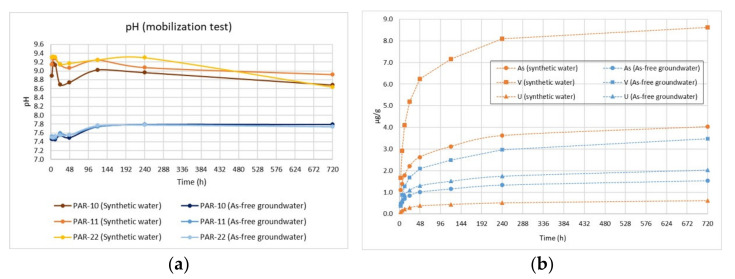
Batch test results. (**a**)—pH monitoring (3 rock samples, mean of two replicates); (**b**)—As, V and U released during the test (sample PAR-11, mean of two replicates).

In Figure 6a, the pH monitoring during the first batch test on the three selected tuffs is shown. The pH values are always higher (8.7–9.3) for the synthetic water than the As-free groundwater, which always shows values closer to neutrality (7.4–7.8) due to buffer capacity. As and V are strongly affected by high pH values and, to a lesser extent, by the presence of other exchanging anions, as observed for U (Figure 6b). Arsenic values are always higher for the synthetic water, reaching a concentration of 202 µg/L (against 77 µg/L for the As-free groundwater), corresponding to 14.9% of As released. The greater As and V release at high pH, compared to near-neutral conditions, is promoted by the alkaline desorption processes.

A batch desorption study by Kim et al. [18] reported that hydrolysis of Fe (hydr-) oxides released As and F under reducing conditions; however, the same study also found that under oxidizing conditions, an increase in pH was the main mode of As and F mobilization. At pH above 8, a significant displacement of arsenate is induced by OH- ions through an anion exchange mechanism.

The second test was divided into different steps, simulating the water-rock interaction processes in oxygenated conditions (STEP 1) under anaerobic conditions and weakly positive redox potential (STEP 2a) and in anoxic and strongly reducing conditions (STEP 2b), with the latter being locally observed in the study area.

**Figure 7:**
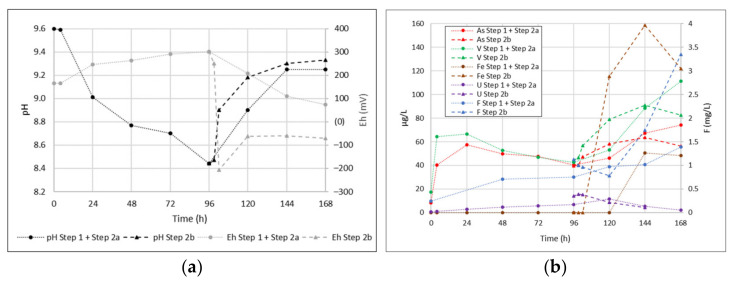
Behavior of different parameters during the second batch test. (**a**)—Eh and pH values; (**b**)—As, V, Fe, U and F concentrations.

Figure 7a shows the trend of pH and Eh values during the second batch test. DO showed stable values around 7.8 mg/L during the aerobic phase, while it suddenly reduces during the second step (complete anaerobic conditions are established 15 min after fluxing with N_2_).

Eh showed a slight and progressive increase in STEP 1, followed by a decrease to slightly positive values in STEP 2a (about +70 mV), while a sharp decrease due to the addition of a reducing species (S^2−^) can be observed in STEP 2b before it stabilized at slightly negative values (about −60 mV). The pH after the water-rock interaction always remains at rather high values (8.4–9.6) and tends to progressively decrease during the aerobic phase and increase in the anaerobic ones, due to N_2_ blowing.

Arsenic and vanadium (Figure 7b) follow a similar trend during the test. The two elements show an increase in the first 24 h during the aerobic phase and then a slight decrease towards the end of this step, probably due to the re-sorption of the oxyanions. The desorption/resorption processes from zeolites cannot be excluded. In the anaerobic step, the concentration of As and V gradually increased and may also have been affected by the dissolution of the iron oxides in an anoxic environment, as shown by the increase in Fe content, which was up to 150 ug/L.

The uranium trend appears to be strongly influenced by pH variations, with an increase in the aerobic phase and then a decrease to 1.1 µg/L at the end of the anaerobic step, suggesting a probable re-adsorption, given the affinity of uranyl for Fe oxide phases [95]. In contrast, F shows a gradual increase of the concentration both in the aerobic and anaerobic steps (only 3 samples in STEP1), thus appearing to be linked to dissolution processes and not influenced by the variable pH-Eh conditions affecting the test.

Regarding STEP2b (reducing conditions), As and V again show a very similar trend, with an increase of concentrations for a large part of the step and a final slight decrease, probably due to the partial precipitation of sulfides. Here, Fe, undetectable for a large part of the test, shows appreciable concentrations and apparently similar behavior. Uranium still appears constrained by the pH increase and tends to the gradual reduction of the concentration towards the end of the test. Fluoride, on the other hand, shows a distinct trend almost opposite to that of As and V, with a slight concentration decrease in the first 24 h and then a marked increase in the following 48. Therefore, the element is strongly mobilized under alkaline and reducing conditions.

### 3.5. Arsenic and Other PTEs: Linking Solid Matrix Analysis with Groundwater Geochemistry

As observed in Table 2, arsenic in groundwater in the northern sector (more representative of the volcanic domain) is well correlated with other PTEs, suggesting common processes. The link between these elements and the release from the volcanic tuff under different experimental conditions was therefore analyzed, investigating possible connections with groundwater data.

Uranium in the two batch tests shows a different behavior in regard to As, appearing strongly linked to pH variations in the frankly alkaline environment. The lack of correspondence between field and laboratory data could therefore be attributed to the different existing conditions, with lower pH values in the study area (especially in the northern sector) than those measured during the tests. However, it should be emphasized that the correlation with As is observable at the low concentrations (median = 0.8 µg/L; max = 13.9 µg/L) found in the northern sector. In the southern area, U, although showing more important concentrations (median = 6.6 µg/L; max = 34.6 µg/L), poorly correlates with As.

The strong As-F correlation is constant in groundwater in both sectors. A correlation is also partially observable in the second batch test, but only in the aerobic/anaerobic steps (1 and 2a). Beyond the common origin of the two elements due to water-rock interaction processes, in the study area, As and F remain strongly linked in aqueous solution, and processes capable of breaking this common mobility do not intervene. On a regional scale, Parrone et al. [96] attributed this stable co-presence to the widespread geochemical background of cold groundwater circulating volcanic formations, also highlighting areas where the good correlation is lost due to localized peculiarities (e.g., in the proximity of fractures/faults systems or mineral deposits).

In contrast, during the batch test, As and F showed almost opposite behaviors in the reducing step (2b). The F concentration shows a noticeable increase, probably due to an exchange with OH at high pH, while As is probably released by reductive processes and partially re-precipitated as sulfide. Reducing conditions are uncommon in the study area but can locally affect the As-F correlation. For example, the well N32, one of the few points having strongly reducing conditions (Eh = −323 mV), shows unusually low concentrations of As and V (0.2 and 2.2 µg/L) and high values of F (1.9 mg/L). This could be due to the mixing of groundwater with reducing deep fluids, leading to the subsequent precipitation of As and V as sulfides and the loss of the As-F correlation. Once eliminated, these local geochemical anomalies, usually characterized by physical-chemical parameters’ abnormal values (negative Eh, low DO, high EC), cause the As-F correlation to further increase, up to a correlation of 0.8. Additionally, Kim et al. [18] identify a good correlation between As and F under oxidizing conditions and increasing pH, associated with the desorption from Fe (hydr)oxides, while a poorer correlation between the two elements verifies in reducing aquifers due to sulfate reduction and consequent As precipitation as sulfide, a process not affecting F concentration.

**Figure 8:**
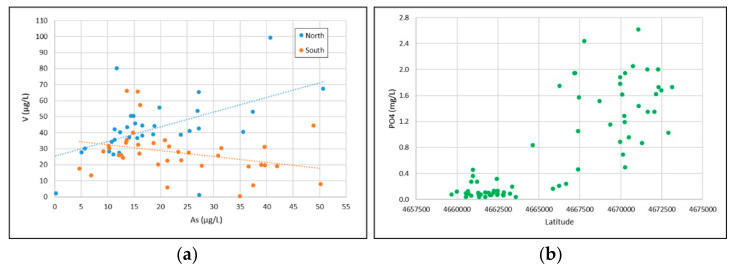
(**a**) scatterplot As-V for the northern and southern sector; (**b**) Latitude trend of the PO_4_ concentrations.

The good As-V correlation observed in groundwater of the northern sector is confirmed by the results of the two batch tests performed on the solid matrix. The two elements show completely overlapping trends in all conditions and therefore can be considered representative of the water-VTYT interaction process. The good correlation in groundwater is lost in the southern sector, where the importance of sedimentary deposits increases. The presence of clays could affect the As-V correlation through sorption/desorption processes. Zhu et al. [97] observed the consistent pH-dependent sorption of V onto kaolinite and montmorillonite, with relatively high sorption capacity occurring at the pH range of 4–10. However, the electrostatic repulsion between the V anions and the negatively charged surface determines the very low maximum adsorption capacities of the two clay minerals (<1.0 mg/g) compared to the result of V adsorption onto soil [98] and soil colloids [99]. This is due to the high surface area of soil colloids and the presence of Fe/Al (hydr)oxides, which have a high tendency for V adsorption [100]. Recently Gonzalez-Rodriguez and Fernandez-Marcos [101] observed high sorption of vanadate and arsenate onto non-crystalline iron and aluminum species. Furthermore, they found that phosphate can induce the desorption of arsenate, while it can hardly displace adsorbed vanadate. This different tendency to displace As and V may be a plausible explanation for the loss of positive correlation in the southern sector (Figure 8a), where PO_4_ indeed shows very low concentrations and may have undergone sorption processes (Figure 8b). This could explain also why As in the southern area remains in solution and maintains its strong correlation with F.

## 4. Conclusions

The analysis of the distribution of PTEs in the different matrices (groundwater, rocks) and the study of the geochemical processes responsible of their mobilization can provide a comprehensive framework on the origin and evolution of geogenic contaminants in groundwater.

The joint analysis of field data and laboratory observations suggests the existence of a diffused As geochemical background due to the water-rock interactions. In many hydrogeological settings, as alluvial aquifers, the release of As has been proved to be triggered by reductive dissolution, while in geothermal contexts, it is often explained by upraising geothermal fluids, mobilization from volcanic rocks due to acidity or high temperature, and local processes as a release from carbonate trapping. Our study pointed out that in volcanic areas, although the reductive dissolution of Fe oxyhydroxides could cause an enhanced localized release of As into groundwater, the anion exchange induced by specific exchangers (e.g., phosphates) with As adsorbed on the surface seems to be the most widely diffused process, which is often not taken into consideration in other studies.

The strong and stable As-F correlation in groundwater already observed at the regional scale has been confirmed, while the As-V correlation is lost during the transition towards the sedimentary deposits, thus representing a good geochemical marker of the volcanic hydrogeological domain.

Investigating PTEs adsorption/desorption behavior at variable pH and Eh conditions could give useful hints for the comprehension of the phenomena that release arsenic in groundwater exploited for human consumption, hence suggesting suitable technology and management options for the distribution of As-free waters in affected areas.

## Figures and Tables

**Table 1:** Selected sequential extraction procedure for As fractioning.

Step	Extractant	Time/Temperature	As Fraction	Reference
1	(NH_4_)_2_SO_4_ 0.05M	4 h/25 °C	Easily exchangeable As	[57]
2	(NH_4_)H_2_PO_4_ 0.05M	16 h/20 °C	Specifically adsorbed As	[57]
3	Acetic buffer 1M (pH 5)	12 h/25 °C	As bound with calcite	[61]
4	NH_4_-oxalate buffer 0.2M (pH 3.2)	4 h/20 °C + 10 min (wash)	Low crystalline Fe(III)-oxyhydroxides	[57]
5	NH_4_-oxalate buffer 0.2M + ascorbic acid 0.1M (pH 3.2)	30 min/96 °C + 10 min (wash)	Crystalline Fe(III)-oxides	[57]
6	HNO_3_ 8M	3 h/80 °C	Sulfides	[84]
7	Microwave digestion (HNO_3_ + H_2_O_2_)	30 min/180 °C	Residual fraction	[57]

**Table 2:** Pearson’s (*r*) and Spearman’s (*r_s_*) correlation coefficients among As and other chemical and physical-chemical parameters. Positive correlations are highlighted on a green scale, negative correlations on a red scale. Statistically significant values in bold (α = 0.05).

Parameter	North		South	
	*r*	*r_s_*	*r*	*r_s_*
Eh	0.083	−0.175	−0.255	**−0.338**
T	0.185	0.109	0.291	0.318
pH	0.291	**0.379**	0.242	0.302
DO	−0.066	−0.040	**−0.618**	**−0.619**
Cond	−0.066	0.183	0.156	0.121
F	**0.658**	**0.690**	**0.741**	**0.805**
Cl	−0.157	−0.121	−0.167	−0.180
PO_4_	−0.107	0.025	−0.223	−0.301
SO_4_	−0.327	−0.264	0.288	**0.449**
HCO_3_	0.165	0.261	0.161	0.161
Na	0.216	0.280	**0.630**	**0.488**
K	0.055	0.194	**0.541**	**0.542**
Mg	−0.250	−0.172	**−0.356**	**−0.449**
Ca	−0.076	0.241	−0.105	−0.225
Al	0.002	0.036	−0.070	0.213
Mn	−0.189	0.028	0.034	0.244
Fe	0.114	−0.040	0.156	−0.013
U	**0.613**	**0.536**	0.230	**0.399**
Li	0.230	0.326	**0.711**	**0.686**
B	**0.366**	0.111	**0.365**	**0.497**
V	**0.533**	**0.574**	**−0.326**	−0.309
Ni	−0.217	−0.041	−0.116	0.011
Cu	−0.192	−0.324	0.083	0.029
Zn	**0.397**	0.060	−0.219	−0.195
Ba	0.045	0.003	−0.161	−0.177

## Data Availability

The data presented in this study are available on request from the corresponding author.

## Supplementary Materials

The following supporting information can be downloaded at: https://www.mdpi.com/article/10.3390/toxics10060288/s1, Table S1: Chemical analysis of the two solutions used for the first experiment; Table S2: Comparison of the main statistics for all the analyzed parameters, distinguished between northern and southern sectors; Figure S1: Distribution of As concentrations in groundwater of the study area.
