# Supplementary material for: Water-Rock Interaction Processes: A Local Scale Study on Arsenic Sources and Release Mechanisms from a Volcanic Rock Matrix
Source: Toxics. 2022 May 27;10(6):288. doi: 10.3390/toxics10060288 (PMC9230518; doi:10.3390/toxics10060288)
Supplement: Supplementary file 1 [file toxics-10-00288-s001.zip › toxics-1727863-supplementary.pdf]

toxics

MDPI

Water-Rock Interaction Processes: A Local Scale Study on Arsenic Sources and Release Mechanisms from a Volcanic Rock Matrix

Daniele Parrone *, Stefano Ghergo, Elisabetta Preziosi and Barbara Casentini

Table S1. Chemical analysis of the two solutions used for the first experiment.

Parameter	As-Free Groundwater	Synthetic Water
pH	7.2	5.7
DO (mg/L)	8.0	8.0
Eh (mV)	210	250
El. Cond. (µS/cm)	634	70
F (mg/L)	0.5	-
Cl (mg/L)	27.7	-
Br (mg/L)	0.05	-
NO_3 (mg/L)	4.9	31.2
PO_4 (mg/L)	0.03	-
SO_4 (mg/L)	49.3	-
HCO_3 (mg/L)	268.5	-
Na (mg/L)	30.0	-
Mg (mg/L)	13.8	-
K (mg/L)	3.9	19.7
Ca (mg/L)	67.5	-
Al (µg/L)	4.0	-
V (µg/L)	0.6	-
Mn (µg/L)	1.5	-
As (µg/L)	0.4	-
Ba (µg/L)	38.8	-
U (µg/L)	1.1	-

Table S2. Comparison of the main statistics for all the analyzed parameters, distinguished between northern and southern sector.

Parameter.	Mean		Median		Min		Max		Std. Dev.	
	North	South	North	South	North	South	North	South	North	South
Eh (mV)	253	207	279	227	-323	-177	546	294	156	82
T (°C)	15.9	17.2	15.7	17.3	14.6	13.1	18.0	20.4	0.9	1.6
pH	6.2	7.7	6.1	7.7	5.4	7.0	7.3	8.6	0.5	0.3
O_2 (mg/L)	5.2	6.3	4.9	7.1	0.1	0.1	9.2	9.7	2.9	2.6
El. Cond. (µS/cm)	618	613	557	605	245	449	2660	1025	440	119
F (mg/L)	1.3	1.8	1.1	1.4	0.3	0.7	3.5	4.0	0.7	0.9
Cl (mg/L)	19.5	33.1	18.7	32.3	10.6	16.4	35.3	67.6	4.9	10.6
NO_3 (mg/L)	16.0	12.0	13.3	11.8	0.7	1.7	50.7	37.2	10.9	7.3
PO_4 (mg/L)	1.50	0.15	1.59	0.10	0.46	0.04	2.62	0.84	0.52	0.15
SO_4 (mg/L)	49.0	17.8	8.8	12.0	3.7	3.7	1018.6	122.6	178.1	21.0
HCO_3 (mg/L)	258.0	274.1	224.5	263.5	36.6	176.9	966.2	434.3	205.7	61.3
Na (mg/L)	26.9	38.5	26.4	34.4	15.2	23.8	59.3	70.6	9.1	12.5
Mg (mg/L)	15.9	10.4	12.0	9.8	4.1	5.5	132.0	19.7	22.2	3.3
K (mg/L)	45.1	40.0	44.1	37.9	12.1	28.4	138.0	57.3	24.5	8.0

Ca (mg/L)	55.3	48.3	41.9	42.1	10.3	29.5	378.0	131.8	66.9	19.6
Li (μg/L)	27.4	26.0	22.3	21.6	5.4	9.6	121.0	73.3	26.5	14.1
B (μg/L)	88.4	93.5	78.5	83.7	37.3	47.8	212.0	256.0	41.9	43.7
Al (μg/L)	88.2	23.8	46.0	3.9	5.8	0.1	1006.0	471.0	175.7	77.6
V (μg/L)	42.6	27.7	40.9	27.1	1.1	0.4	99.4	66.2	18.8	14.1
Cr (μg/L)	0.9	1.0	0.9	1.1	0.2	0.4	1.9	2.1	0.5	0.4
Mn (μg/L)	102.9	12.8	4.1	0.7	0.2	0.1	1904.2	282.0	346.9	47.0
Fe (μg/L)	160.6	84.7	15.4	17.1	5.8	0.9	3953.0	887.2	696.6	194.4
Ni (μg/L)	1.1	0.4	0.7	0.2	0.2	0.1	5.3	2.2	1.4	0.5
Cu (μg/L)	1.9	0.8	0.8	0.4	0.2	0.1	13.5	4.5	2.6	1.0
Zn (μg/L)	26.4	16.5	7.2	9.6	1.1	0.5	256.0	88.1	55.0	19.9
As (μg/L)	18.8	23.0	15.4	20.8	0.2	4.7	50.6	50.2	11.0	12.2
Se (μg/L)	0.2	0.3	0.2	0.2	0.2	0.1	0.3	1.1	0.0	0.2
Ba (μg/L)	55.9	58.4	44.5	54.8	8.3	1.6	193.0	155.0	44.5	29.9
U (μg/L)	1.8	9.8	0.8	6.6	0.2	0.9	13.9	34.6	2.6	9.3

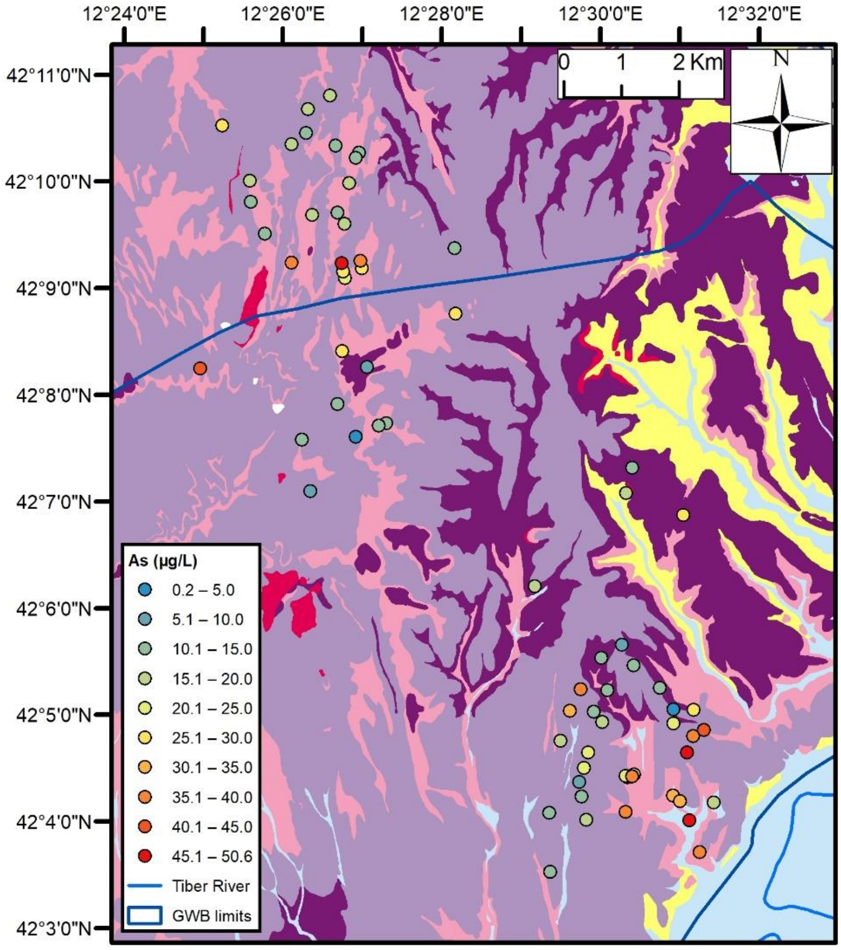

Figure S1. Distribution of as concentrations in groundwater of the study area.
